# Proteome diversification by genomic parasites

**DOI:** 10.1186/s13059-016-0875-6

**Published:** 2016-01-30

**Authors:** Eli Eisenberg

**Affiliations:** School of Physics and Astronomy, Raymond and Beverly Sackler Faculty of Exact Sciences, Tel Aviv University, Tel Aviv, 69978 Israel

## Abstract

*Alu* elements can integrate into human genes and lead to the generation of primate-specific isoforms. A new study examines their contribution to the human proteome.

Please see related Research article: http://www.genomebiology.com/2016/17/1/15

An estimated 2000 human exons have been generated from the inclusion of primate-specific *Alu* elements into mRNA transcripts. These *Alu* exons are extensively transcribed, but the prevailing view in recent years has been that they are rarely translated into proteins. A recent article published in *Genome Biology* by Lin and colleagues [1] challenges this prevailing view and reports new findings that highlight the important contribution of *Alu* exons to the human proteome.

## Junk, active junk, useful junk

It has been known for many years that a sizable fraction of the genomes of higher organisms comprises 'junk DNA', which are sequences that have little or no effect on phenotype(s). This notion has been often supported by the observation that protein-coding sequences account for a small percentage of the genomes of many organisms, and in humans this figure is only ~1.5 %. However, it has been repeatedly demonstrated in recent decades that 'non-coding' should not be confused with 'non-functional'. Many non-coding DNA regions are highly conserved and have important functional roles. There are two different ways by which non-coding DNA could be functional: the first is where it is transcribed into non-coding functional RNA, such as ribosomal RNAs and micro-RNAs; the second is where non-coding DNA is not transcribed but still has an important role, such as DNA regions that bind proteins or RNA, thus leading to enhancement or inhibition of transcription of an adjacent gene. Thus, some of the non-coding DNA is definitely functional, but how much of it?

Nearly half of the human genome can be identified as being derived from transposable elements, which are parasitic pieces of DNA that self-replicate and proliferate in the host genome [2]. As a result, the human genome harbors a number of families of short segments that are repeated hundreds of thousands of times. The vast majority of these copies are mutated to a point where they are no longer able to replicate, but remain in the genome as evidence of past retroelement activity. The low information content of these millions of repeated genomic regions, and their apparent neutral evolution, naturally led to the assumption that they must be, by and large, useless. These ‘dead’ transposable elements would thus be a classic example of junk DNA, encompassing almost half of our genome.

This notion was apparently challenged by results from the Encyclopedia of DNA Elements (ENCODE) project that showed that more than 75 % of the human genome is transcribed under some conditions, and at least 80 % of the human genome participates in some biochemical RNA-associated and/or chromatin-associated activity [3]. That is, the presumed junk DNA, including repetitive elements, is apparently not as neutral as previously thought, and does take part in various biochemical activities. This was interpreted by many as the death of the junk DNA concept [4]. However, the results, and their interpretation, were fiercely criticized [5]. It was pointed out that selective pressure might affect less than 15 % of the human genome. This estimate is likely to be conservative, but it does suggest that while most, if not all, of the human genome is biochemically active, a large part of this activity might be evolutionarily neutral—’biological noise‘. In other words, claim the critics, biochemically active junk is still, after all, nothing but junk.

The debate over the relative extent of biological noise versus functional activity is ongoing, and to a large extent it boils down to the semantic question of what is meant by 'functional' [3]. However, it is certainly accepted that biochemically active regions might have been exapted, that is utilized for a function, to participate in various biological activities that are functional by all means. If exapted regions are human-specific or primate-specific, they might appear as selectively neutral. Identifying these cases of active junk turning into useful junk is a major challenge.

## Exonization of *Alu* repeats

One particularly dramatic example of exaptation in the case of retroelements is the process of exonization; this is where a genomic sequence originating from retrotransposon activity is integrated into a gene-encoding locus, which leads to the formation of a new exon and its addition to a coding sequence [2, 6]. These newly formed exons are often alternatively spliced, such that a novel splice-variant including the retroelement exon can be translated alongside the original protein-coding sequence.

The molecular mechanisms by which *Alu* elements lead to the exonization of intronic sequences have been studied in detail for the *Alu* elements [6]. *Alu* elements are primate-specific retrotransposons that account for over 10 % of the human genome, with over one million copies known. They are non-autonomous transposable elements, their sequence does not code for proteins, and they replicate by acquiring *trans*-acting factors. *Alu* exonization is facilitated by the fact that *Alu* elements are abundant in introns, and the *Alu* consensus sequence harbors multiple sites that resemble splice sites. Accordingly, the number of mutations leading to *Alu* exonization is surprisingly small. Therefore, *Alu* elements are frequently exonized to create new, primate-specific exons; approximately 2000 human exons are *Alu*-derived, making the *Alu* element a major contributor to lineage-specific new exons in the primate and human genomes. Furthermore, while *Alu* exons are typically alternatively spliced, a significant proportion of them have high transcript inclusion levels, or tissue-specific splicing profiles. The recent *Genome Biology* study by Lin and colleagues identified 52 such putative coding exons [1]. Alu exons are therefore prime candidates for having important regulatory roles in modulating mRNA degradation by nonsense-mediated decay (NMD), or affecting translational efficiency of mRNA transcripts. Thus, they have the potential to confer human-specific or primate- specific gene regulation, and corresponding phenotypes.

## Do *Alu* repeats diversify the human proteome?

The coding capacity of *Alu* exons, or, more generally, the extent to which transposable-element sequences encode host proteins, has been a controversial topic over the past decade [7]. Transcriptome-based methods have led to an estimate of 4 % of the human coding sequences harboring transposable elements; however, conservative methods focusing on well-characterized proteins or using proteomic sequence methods resulted in a much lower number of ~0.1 %. In particular, it was claimed that non-autonomous transposable elements that do not encode any protein, such as *Alu* elements, are unlikely to provide protein-coding sequences. Thus, although *Alu* elements are frequently exonized into protein-coding sequences, the prevailing view deemed their capacity for proteome diversification to be minute.

Lin and colleagues [1] have now re-examined this issue by employing a new tool—they mined the large-scale mass-spectrometry PRIDE dataset to look for evidence for proteins resulting from *Alu* exons. They analyzed 911 *Alu* exons located within gene coding sequences and found that 262 (29 %) of those would not trigger transcript degradation through mRNA NMD. Notably, the peptide database provided evidence for the translation of one-third of putative coding *Alu* exons into proteins (85/262). This included 18 of the 52 putative protein-coding *Alu* exons with RNA-seq evidence for high splicing activity in human tissues.

Although the absolute number of *Alu* exons Lin and colleagues [1] reported to be translated is not too large, the recovery fraction—one-third of the putative coding exons—is striking when compared with that of similar studies. For example, a recent study searching for peptide evidence of novel splice junctions recovered only 0.5 % of the well-covered splice junctions predicted from RNA-seq [8]. Moreover, the actual number of coding *Alu* exons is likely to be even higher than that reported by Lin and colleagues [1]. First, mass-spectrometry proteomic methods are still far from perfect, and the limited dynamic range of their analysis is still an issue [8]. Additionally, another intriguing scenario related to the phenomenon of extensive RNA editing of *Alu* sequences should be considered. Millions of adenosine sites within *Alu* sequences can be targeted by the ADAR enzymes and deaminated into inosines, which are interpreted by cellular machinery as guanosines [9]. Thus, as Lin and colleagues point out [1], *Alu* exons that seem to be putatively non-coding, owing to an apparent NMD signal, might actually code for proteins provided that RNA editing removes the premature stop codon from the transcript. Such a scenario has already been demonstrated for the *NARF* gene (encoding nuclear prelamin A recognition factor), where RNA editing both creates a splice site and removes a stop codon in an *Alu* exon [10]. Indeed, peptides supporting translation were reported for 47 out of 649 NMD-inducing exons. Many of these could very well be false positives, thus setting a rather conservative bound on the false detection level, but the possibility of dozens of editing-dependent coding exons is fascinating.

These results shed a new light on the process of proteome diversification by retroelements. The crucial step for *Alu* exaptation, it seems, is exonization. Only one in a thousand *Alu* repeats was located in the right position and accumulated the required mutations to make it exonized into the coding sequence. Once exonized, approximately one-third of the cases result in a putative coding sequence, and at least one-third of these actually gets translated. Possibly, the latter stages of this process involve some additional mutations, consistent with the observation that the younger *Alu*-Y exons, even if putatively coding, are less likely to produce proteins.

## Junk turned useful?

Assessing the functionality of *Alu* exons remains an open question. The demonstration that *Alu* exons produce novel proteins is a significant advance, but is not sufficient to deem these DNA regions as having a function that provides a selection advantage. In as much as not all non-coding DNA is junk DNA, not all coding DNA is necessarily useful. It is still important to show how novel *Alu* proteins bring about unique functionality that provides a selective advantage. For one particularly insightful example—the RNA editing enzyme ADARB1—it has previously been shown that inclusion of a peptide derived from an exonized *Alu* affects the editing activity. Lin and colleagues further show that, in some cases, the site selectivity of ADARB1 also differs between the protein products [1]. However, further work is still required in order to understand whether the modified protein activity due to *Alu* peptide inclusion in ADARB1, or any gene for that matter, was indeed exapted to provide humans with a selective advantage.

In summary, although the vast majority of retroelements are likely to be selectively neutral, the relatively few that have been exonized, effectively spliced and translated to create novel protein products are much more likely to have had an impact on primate evolution.

## Abbreviations

ENCODE: Encyclopedia of DNA Elements
NMD: nonsense-mediated decay
